# Loss of Multimerin-2 and EMILIN-2 Expression in Gastric Cancer Associate with Altered Angiogenesis

**DOI:** 10.3390/ijms19123983

**Published:** 2018-12-11

**Authors:** Eva Andreuzzi, Alessandra Capuano, Rosanna Pellicani, Evelina Poletto, Roberto Doliana, Stefania Maiero, Mara Fornasarig, Raffaella Magris, Alfonso Colombatti, Renato Cannizzaro, Paola Spessotto, Maurizio Mongiat

**Affiliations:** 1 Dipartimento della Ricerca e della Diagnostica Avanzata dei Tumori, Divisione di Oncologia Molecolare, Centro di Riferimento Oncologico di Aviano (CRO) IRCCS, 33081 Aviano, Italy; eandreuzzi@cro.it (E.A.); acapuano@cro.it (A.C.); rpellicani@cro.it (R.P.); evelianapoletto@gmail.com (E.P.); rdoliana@cro.it (R.D.); acolombatti@cro.it (A.C.); pspessotto@cro.it (P.S.); 2 Dipartimento di Oncologia Clinica, Gastroenterologia Oncologica Sperimentale, Centro di Riferimento Oncologico di Aviano (CRO) IRCCS, 33081 Aviano, Italy; smaiero@cro.it (S.M.); mfornasarig@cro.it (M.F.); raffaella.magris@cro.it (R.M.); rcannizzaro@cro.it (R.C.)

**Keywords:** tumor microenvironment, extracellular matrix, angiogenesis, lymphangiogenesis, gastric cancer, endomicroscopy

## Abstract

Gastric cancer is a deadly tumor and a relatively common disease worldwide. Surgical resection and chemotherapy are the main clinical options to treat this type of disease, however the median overall survival rate is limited to one year. Thus, the development of new therapies is a highly necessary clinical need. Angiogenesis is a promising target for this tumor type, however clinical trials with the use of anti-angiogenic drugs have so far not met expectations. Therefore, it is important to better characterize the expression of molecules whose expression levels may impact on the efficacy of the treatments. In this study the characteristics of the gastric tumor associated blood vessels were first assessed by endomicroscopy. Next, we analyzed the expression of Multimerin-2, EMILIN-2 and EMILIN-1, three molecules of the EMI Domain ENdowed (EDEN) protein family. These molecules play important functions in the tumor microenvironment, affecting cancer progression both directly and indirectly impinging on angiogenesis and lymphangiogenesis. All the molecules were highly expressed in the normal mucosa whereas in a number of patients their expression was altered. We consider that better characterizing the gastric tumor microenvironment and the quality of the vasculature may achieve effective patient tailored therapies.

## 1. Introduction

Gastric cancer is the third leading cause of cancer-related deaths and a relatively common malignancy worldwide [1]. Surgical resection of the tumor represents the adopted option to improve the chances of long-term cure; however most of the patients at diagnosis are characterized by a locally advanced or metastatic disease. To improve the lifespan, these patients are subjected to palliative chemotherapy, which includes cisplatin, docetaxel, oxaliplatin and 5FU, among other drugs [2,3,4]. Unfortunately, advanced or metastatic gastric cancer is a deadly disease with less than a 10% survival rate at 5 years from the diagnosis, with the median overall survival (OS) being restricted to only 1 year [5].

Hence, given the poor outcome of these patients, the development of new more specific targeted therapies is a highly awaited clinical need. Mutational analysis may offer the possibility to improve the survival rate through the use of targeted drugs, however the targeting of oncogenic mediators such as epidermal growth factor receptor (EGFR), mTOR or c-Met was proven not to be successful in a number of clinical trials [6,7,8,9,10,11,12]. Improvement of progression free survival (PFS) and OS was observed following chemotherapy combined with trastuzumab in patients displaying positivity for the human epidermal receptor 2 (HER-2), however these tumors only represent a minor portion of gastric cancers [13,14,15]. Targeting the tumor microenvironment is an emerging strategy for the treatment of gastric cancer patients [16] and one important component in this context is the vasculature. Angiogenesis, the development of new blood vessels from pre-existing vasculature, is known to influence tumor growth as well as metastatic dissemination, represents a promising field of investigation and in the latest years has gained attention in gastric cancer research [17,18]. Angiogenesis is regulated by different cell types and is stimulated by the secretion of cytokines and growth factors and also by extracellular matrix (ECM) constituents [19,20]. The angiogenic process is switched on during tumor growth to support the restless growth of tumor cells with nutrients and oxygen [21,22]. Thus, the inhibition of this process has been regarded as an encouraging mean to halt tumor growth. In addition to strategies aimed at suppressing the development of new blood vessels, a new promising alternative is to readdress the aberrant tumor-associated vessels, inducing the formation of a normalized and more efficient vasculature. This could allow a proficient delivery of drugs within the tumor, improving the therapeutic outcome of the patients [23,24,25,26]. One of the major regulators of angiogenesis is the VEGFA/VEGFR2 signaling axis [27,28,29]. Interestingly, high VEGFA levels have been associated with reduced survival of gastric cancer patients and with increased tumor aggressiveness [30,31].

Thus, in order to develop new strategies to target gastric cancer angiogenesis, it is of major importance to better characterize the vasculature associated with this tumor type as well as its microenvironment. To achieve this aim in this study we evaluated the expression of Multimerin-2, a new blood vessels’ maker [32]. Multimerin-2 is an extracellular molecule belonging to the EDEN family [33,34,35]. This family of molecules affects tumor growth playing multifaceted direct and indirect functions [34,36,37,38,39,40]. Our group was the first to describe the angiostatic function of Multimerin-2, exerted, at least in part, through the sequestration of VEGFA and the inhibition of the VEGFA/VEGFR2 signaling pathway [41,42,43]. More recent studies demonstrated a tight interaction of this molecule with endothelial cells and pericytes via C-type lectin molecules [44,45,46,47]. Since the loss of Multimerin-2 expression may affect the properties of the vessels and, consequently, the antiangiogenic therapy efficacy, we consider these analyses may be useful to identify the patients that may benefit from anti-angiogenic therapy. Similarly, the EDEN protein EMILIN-2 modulates angiogenesis activating IL-8 expression [38,40] and affects tumor growth via the modulation of different signaling pathways [36,37,38,39]. The analysis of its expression has been elusive due to the lack of appropriate antibodies and in this study we report for the first time the distribution of the molecule in gastric tissues. The altered expression of EMILIN-2 may also potentially affect the efficiency of the vessels and thus chemotherapy efficacy. Finally, the EDEN protein EMILIN-1 regulates blood pressure [48,49], cell proliferation [50] and has been linked to the regulation of lymphangiogenesis [51,52,53]. Very recently, Chen et al., using a weighted gene co-expression network analysis of other several proteins, identified EMILIN-1 as a candidate gene whose expression levels correlated with pathologic T stage and histologic grade, and also affected the overall survival of gastric cancer patients [54]. However the histological validation and localization of EMILIN-1 in gastric tissues were not known prior to this study. We consider that the analyses reported in this investigation may be useful to better characterize the gastric tumor microenvironment and identify the molecular mechanisms involved in drug resistance in order to achieve more efficacious patient-tailored treatments.

## 2. Results

### 2.1. Gastric Tumors Display a High Angiogenic Score

**Figure 1:**
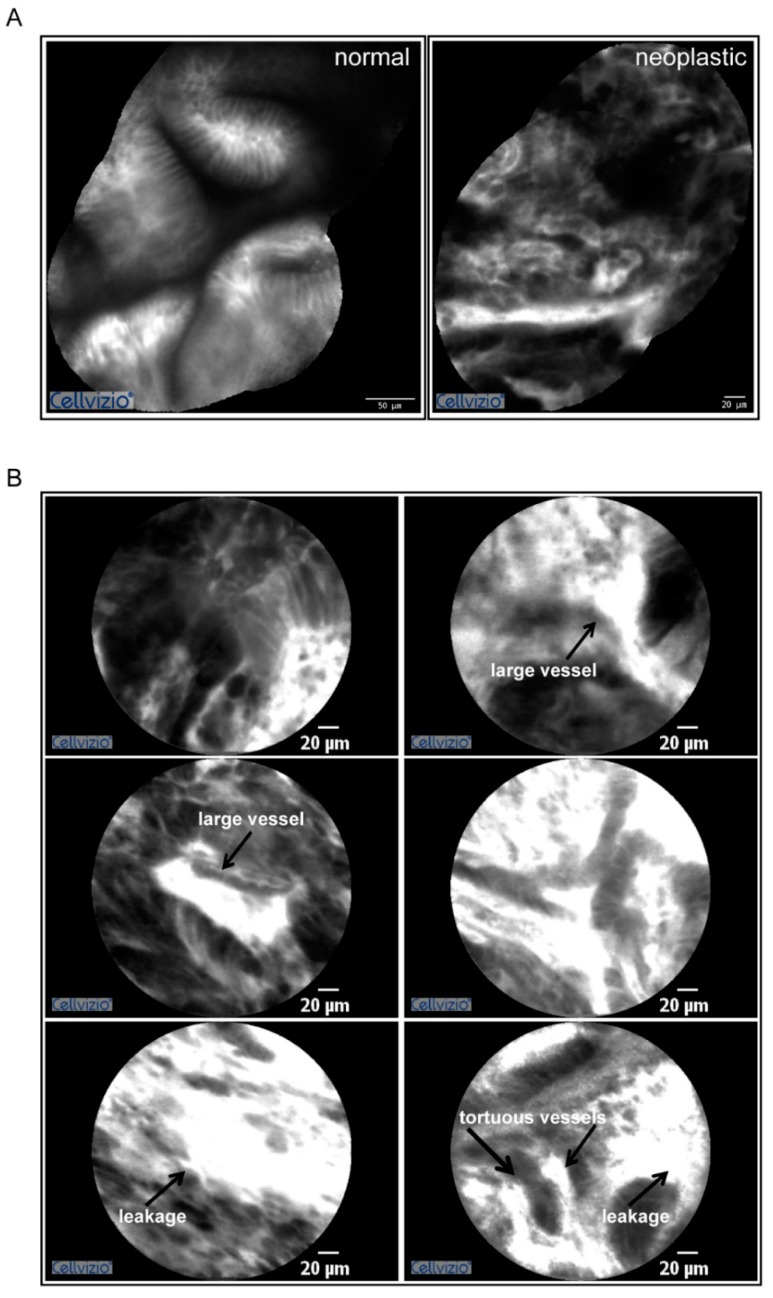
Gastric cancer display a high angiogenic score. (**A**) Mosaic reconstructions of the pCLE analyses of the normal (left) and neoplastic (right) mucosa in a gastric cancer patient characterized by an angiogenic score of 3. (**B**) Representative images captured during pCLE endomicroscopy showing the presence of blood vessels’ leakage, tortuous vessels, and large dilated vessels well detectable in highly vascularized areas. These vessels often display defective blood flow (see Supplemental Video S1).

In this study we analyzed the properties of the gastric tumor microenvironment. We combined a pioneering in vivo approach to evaluate the gastric cancer vasculature with innovative biomarkers analyzed by immunofluorescence on the tumor sections. To this end patients affected by gastric cancer were injected with fluorescein and tumor vascularization was assessed by probe-based confocal laser endomicroscopy (pCLE) during the gastric endoscopy procedure. As previously demonstrated this procedure allows the in vivo evaluation of different parameters affecting vascular efficiency such as vessel diameter, tortuosity, leakiness and vessels defective flow [55,56]. The results of the analyses performed on a representative patient are reported in Figure 1 and the relative video in Supplemental Video S1. Overall most of the gastric cancer patients taken into account displayed a high angiogenic score according to the Cannizzaro/Spessotto scale [55,56], which correlated to the advanced stage of the disease. The gastric tumor stage was T3 in almost all the patients analyzed.

### 2.2. Multimerin-2 and EMILIN-2 Are Differentially Expressed in Gastric Tumors Compared to the Normal Counterpart

**Figure 2:**
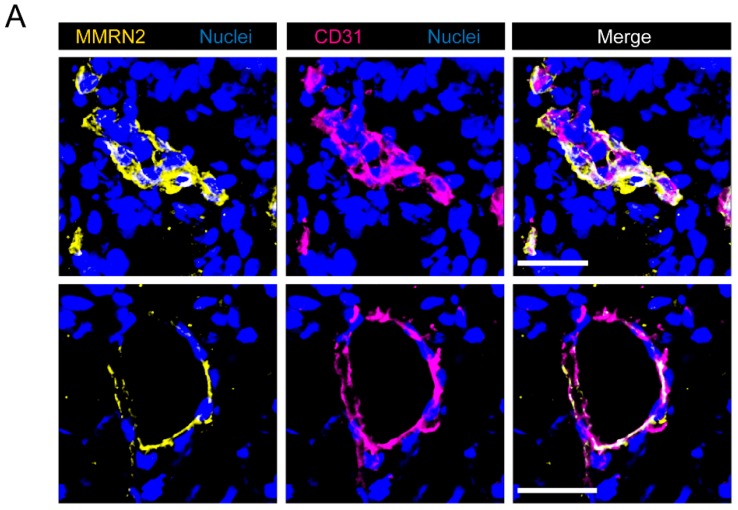

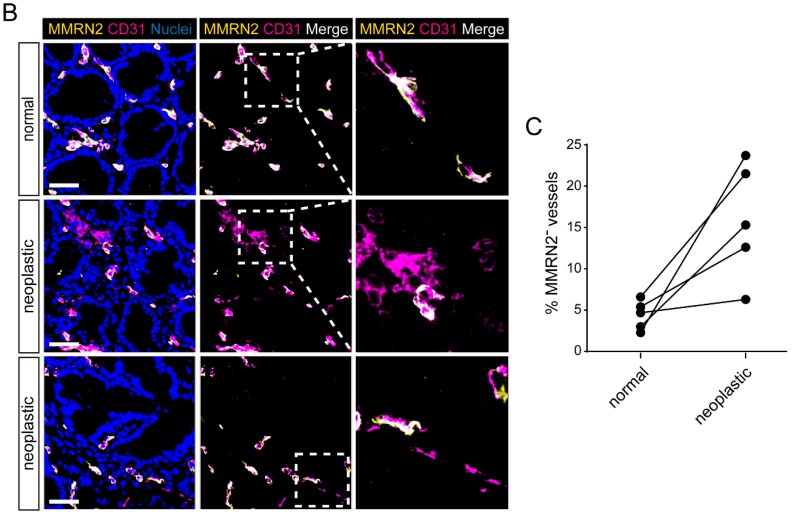
Multimerin-2 expression is lost in a number of gastric cancer-associated vessels. (**A**) Representative images of normal gastric mucosa samples stained with anti-Multimerin-2 (yellow) and anti-CD31 (magenta) antibodies. Nuclei were stained with TO-PRO^®^ 3 (blue). Scale bar = 30 μm. (**B**) Representative images showing the colocalization of Multimerin-2 and CD31 in normal gastric mucosa and in gastric cancer samples. Staining were performed as in A. Scale bar = 50 μm. Magnifications (2.7×) are shown in the panels on the right. (**C**) Graph representing the percentage of blood vessels displaying no staining for Multimerin-2 (MMRN2^−^) in normal and neoplastic samples of 5 patients evaluated by IF as in B.

**Figure 3:**
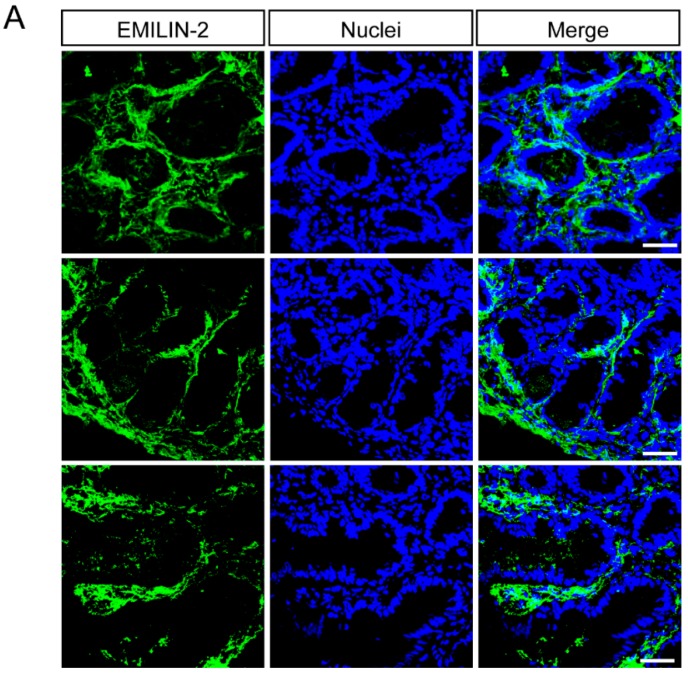

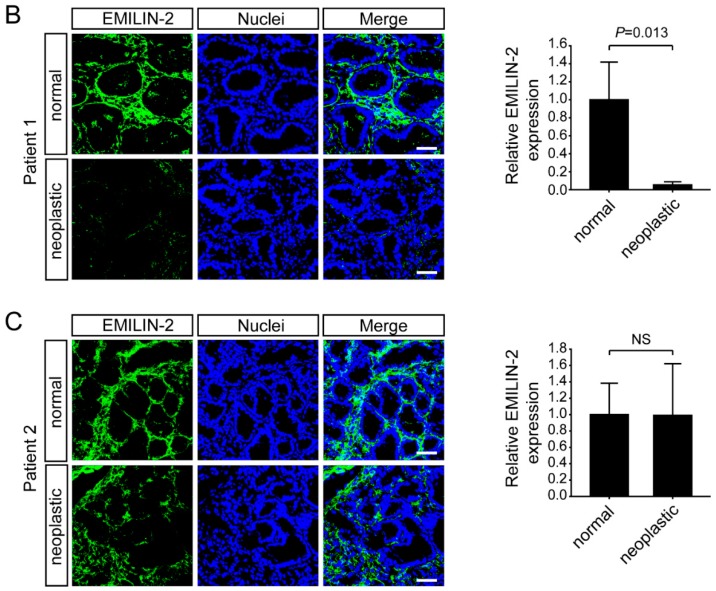
EMILIN-2 expression is decreased in some gastric cancer patients. (**A**) Representative images of normal gastric mucosa samples stained with anti-EMILIN-2 (green). Nuclei were stained with TO-PRO^®^ 3 (blue). Scale bar = 50 μm. (**B**) Images and quantification of the expression of EMILIN-2 (green) in normal and in gastric cancer samples from Patient 1. Nuclei were stained with TO-PRO^®^ 3 (blue). Scale bar = 50 μm. (**C**) Representative images and quantification of the expression of EMILIN-2 (green) in normal and in gastric cancer samples from Patient 2. Nuclei were stained with TO-PRO^®^ 3 (blue). Scale bar = 50 μm. Graphs represent the mean ± SD; *p* values were obtained using the paired Student’s *t*-test. NS: not significant.

We next moved to the analyses performed on the biopsies collected from normal and neoplastic tissues during the gastric endoscopy procedure. We first focused our attention on two of molecules belonging to the EDEN protein family [35] and involved in the regulation of tumor growth and angiogenesis. In particular, we analyzed the expression of Multimerin-2 a glycoprotein expressed by endothelial cells displaying angiostatic functions [20,41,42,43,47] and EMILIN-2, known to affect tumor growth both directly [36,37,38,39] and indirectly impinging on angiogenesis [38,40]. Multimerin-2 expression is confined along the blood vessels and the analysis carried out in this study indicated that gastric vessels also displayed a strong expression of Multimerin-2 which was overlapping with that of CD31, used as a maker to highlight the vasculature (Figure 2A). Interestingly, some of the tumor samples displayed loss of Multimerin-2 expression (Figure 2B,C). However, compared to other tumor types analyzed (data not shown) the loss of Multimerin-2 expression was detected only in a moderate number of vessels (Figure 2C). The same samples were also analyzed for EMILIN-2 expression. These analyses allowed us to identify EMILIN-2 as an important component of the gastric mucosa, the expression being confined to lamina propria (Figure 3A). The expression in the normal tissues was relatively intense, whereas tumoral tissues displayed variable levels of EMILIN-2 expression. In fact, while some of the tumor samples were characterized by EMILIN-2 levels comparable to those of the normal tissue (Figure 3B), other patients displayed a major decrease of EMILIN-2 expression (Figure 3C). In line with our previous results indicating an important role of EMILIN-2 in angiogenesis [38,40], lower levels of EMILIN-2 associated with a significant decrease of CD31 staining only in patient 1, characterized by a higher loss of EMILIN-2 expression (Figure S1). Whereas patient 2, in which the expression of EMILIN-2 was not decidedly altered, did not display a significant decrease of CD31 staining.

### 2.3. EMILIN-1 and EMILIN-2 Are Co-Expressed in the Gastric Lamina Propria

**Figure 4:**
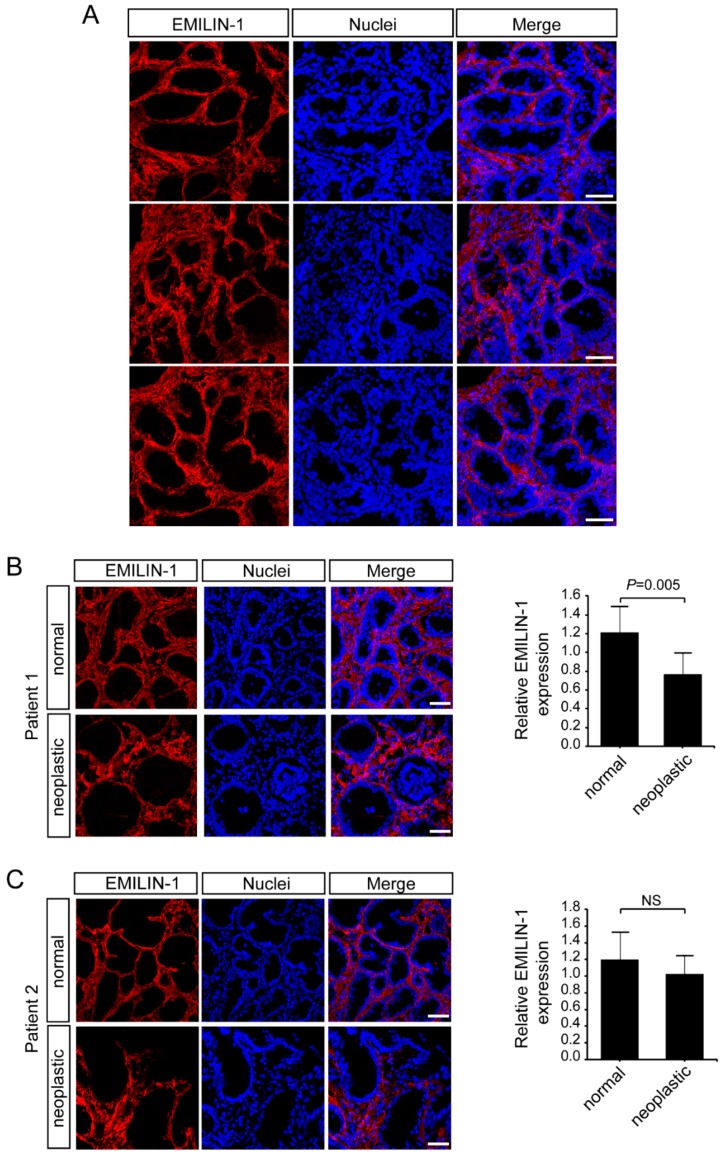
EMILIN-1 deposition is altered in a number of gastric cancer patients. (**A**) Representative images of normal gastric mucosa samples stained with anti-EMILIN-1 (red). Nuclei were stained with TO-PRO^®^ 3 (blue). Scale bar = 50 μm. (**B**) Representative images and quantification of the expression of EMILIN-1 (red) in normal and gastric cancer samples from Patient 1. Nuclei were stained with TO-PRO^®^ 3 (blue). Scale bar = 50 μm. (**C**) Representative images and quantification of the expression of EMILIN-1 (red) in normal and gastric cancer samples from Patient 2. Nuclei were stained with TO-PRO^®^ 3 (blue). Scale bar = 50 μm. Graphs represent the mean ± SD; *p* values were obtained using the paired Student’s *t*-test.

**Figure 5:**
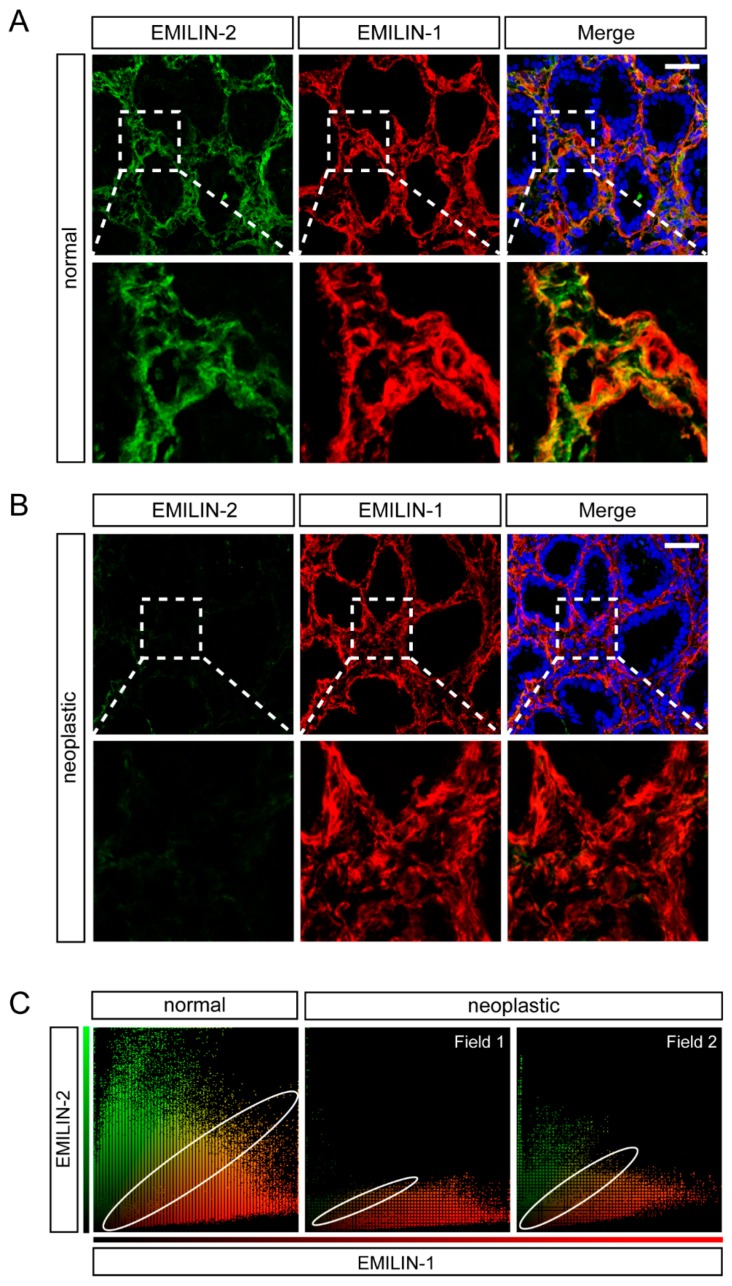
EMILIN-2 and EMILIN-1 expression partially co-localizes in the gastric lamina propria. (**A**) Representative images of the distribution of EMILIN-2 (green) and EMILIN-1 (red) in the normal gastric mucosa. Nuclei were stained with TO-PRO^®^ 3 (blue). Scale bar = 50 μm. Magnifications (3.8×) are shown in the bottom panels. (**B**) Representative images of the distribution of EMILIN-2 (green) and EMILIN-1 (red) in gastric cancer samples. Nuclei were stained with TO-PRO^®^ 3 (blue). Scale bar = 50 μm. Magnifications (3.8×) are shown in the bottom panels. (**C**) Scatter plots from the analysis of EMILIN-2 (green) and EMILIN-1 (red) co-localization in normal and gastric cancer samples as evaluated with the Volocity software. The extent of co-localization (yellow dots) is highlighted by an ellipse.

Next, we analyzed the expression of EMILIN-1. Also in this case, for the first time we show a high deposition of this molecule in the lamina propria (Figure 4A). These analyses indicated that EMILIN-1 levels did not change considerably in the tumor samples (Figure 4B), despite the organization of the molecule was altered in some samples (Figure 4C). Indeed, only one patient with a T1b stage displayed lower EMILIN-1 levels in the tumor compared to the adjacent normal mucosa, whereas EMILIN-1 expression did not change in patients with a T3 stage grading (Figure 4B,C and data not shown). These results were partially in line with recent data showing that EMILIN-1 was expressed at a low level in T1 stage or grade 1–2 patients and at a high level in T2–4 or grade 3 patients [54]. Given that both EMILIN-2 and EMILIN-1 were deposited in the same district, we next simultaneously analyzed the expression of the two molecules both in normal and neoplastic gastric samples. As shown in Figure 5, the expression of EMILIN-2 and EMILIN-1 partially overlapped in normal samples, however EMILIN-1 expression was more adjacent to the epithelium of the gastric glands. Interestingly, when EMILIN-2 expression was strongly decreased in gastric tumor samples, the organization of the EMILIN-1 fibers was also altered (Figure 5B).

### 2.4. Multimerin-2 and EMILIN-2 Affect EC Tubulogenesis in the Context of Gastric Cancer

**Figure 6:**
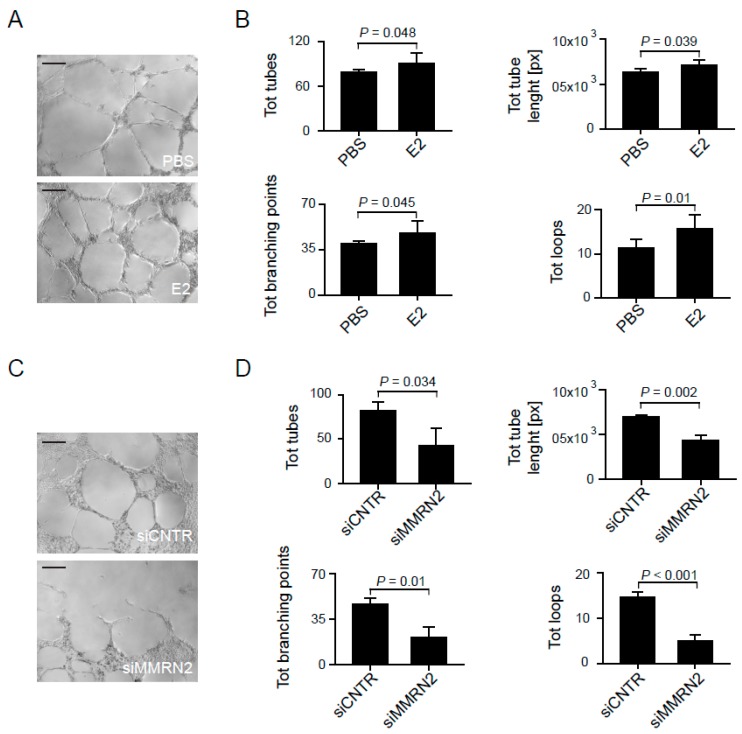
Multimerin-2 and EMILIN-2 expression impact on the angiogenic response in the gastric cancer environment. (**A**) Representative images of the tubes formed by HUVEC cells placed on MATRIGEL^®^ and challenged with conditioned media from AGS cells following the addiction of recombinant EMILIN-2 (E2) or an equal volume of PBS; scale bar = 100 μm. (**B**) Quantification of the total of tubes, total branching points, total tubes length and total loops of the experiment reported in A; [px]: pixels. (**C**) Representative images of the tubes formed by HUVEC cells placed on MATRIGEL^®^ and challenged with conditioned media from AGS following transduction with the control (siCNTR) or Multimerin-2 specific (siMMRN2) siRNA adenoviral constructs; scale bar = 100 μm. (**D**) Quantification of the total tubes, total branching points, total tubes length and total loops of the experiment reported in C. Graphs represent the mean ± SD; *p* values were obtained using the paired Student’s *t*-test.

To verify if the two molecules, whose expression was significantly altered in tumor samples, impacted on angiogenesis in the context of gastric cancer, we carried out tube formation assays on matrigel. To this end, we employed conditioned media from AGS gastric cancer cells. Since EMILIN2 is expressed by different cell types but not by endothelial cells as we have previously shown [40], to verify the role of the molecule in this context we challenged HUVEC cells with an AGS conditioned medium either containing or not containing recombinant EMILIN-2. The presence of the molecule significantly improved tube formation on matrigel in terms of total of tubes, total branching points, total tubes length and total loops (Figure 6A,B). On the contrary, the silencing of Multimerin-2 through a siRNA approach significantly impaired the formation tubes of HUVEC cells placed on matrigel and challenged with conditioned media from AGS cells (Figure 6C,D). In conclusion, in this study for the first time we characterize the expression of Multimerin-2, EMILIN-2 and EMILIN-1 in normal and gastric mucosa and consider that the relative levels of their expression in gastric patients may affect angiogenesis and, possibly, therapy efficacy.

## 3. Discussion

In this study we have focused our attention on the analysis of the expression of ECM molecules of the EDEN protein family [35]. In particular, Multimerin-2 and EMILIN-2 affect angiogenesis and vascular efficiency [38,40,41,42,43,47], whereas EMILIN-1 affects lymphangiogenesis [51,52,53,57], which may also play a role in gastric cancer development representing an important route for metastatic dissemination [58]. Our analyses revealed that the normal gastric mucosa is characterized by a high expression of Multimerin-2 along the blood vessels, whereas the expression of EMILIN-2 and EMILIN-1 was confined to the lamina propria. In particular, we have found that Multimerin-2 co-localizes with virtually all CD-31 positive vessels as previously demonstrated in other tissues [32,47]. On the other end, in gastric tumor samples some of the CD31-positive vessels did not show staining for Multimerin-2. We have previously demonstrated that the VEGFA-driven angiogenic stimulus reduces Multimerin-2 expression [42], thus the loss of Multimerin-2 in gastric tumor vessels may be due to the high levels of VEGFA often found in gastric tumors [31]. It is also possible that the loss of Multimerin-2 may be the result of the activation of MMP-9 and MMP-2, which are activated during active angiogenesis and are known to target Multimerin-2 for degradation [42]. Since loss of Multimerin-2 is associated with an impairment of vascular efficiency (submitted paper), it is plausible to speculate that a high rate of Multimerin-2-devoid vessels could be associated with a worse prognosis for gastric patients. It is known that the efficiency of the vessels is important to grant an optimal delivery of the drugs to the tumors, which is indispensable to achieve therapeutic efficacy. Indeed the knockdown of Multimerin-2 impaired tube formation on MATRIGEL^®^ in the presence of conditioned media from gastric cancer cells. Thus it is conceivable that proper deposition of Multimerin-2 by endothelial cells is necessary to maintain the stability of endothelial cell tubes. Interestingly, compared to EMILIN-1, the levels of EMILIN-2 expression in normal mucosa were lower, as detected in other tissues (unpublished results), despite both the molecules nicely decorating the lamina propria among the gastric glands. However, the levels of EMILIN-2 were considerably higher compared to other tissues analyzed, such as the colon (data not shown). This suggests that EMILIN-2 may be important for the regulation of the gastric function. Interestingly, the analyses of the tumor samples revealed that, while in some of the patients the levels of EMILIN-2 were comparable to the normal counterpart, in other patients the expression was dramatically decreased. In our in vitro studies we showed that EMILIN-2 favored tube formation in the context of gastric cancer. It is possible that this could depend on an increased production of IL-8, as we have recently demonstrated [40]. Thus, also in this case the loss of EMILIN-2 expression may significantly impact on angiogenesis and, as a consequence, on the efficacy of anti-angiogenic therapy. In future studies it would be interesting to determine if the expression of Multimerin-2 and/or EMILIN-2 correlate with the response of patients subjected to anti-angiogenic therapy. Analyses on a higher number of samples will be necessary to determine if these molecules can function as markers to predict the patients’ outcome. In addition, since EMILIN-2 modulates the Wnt/β-catenin signaling pathway [37] which affects the behavior of gastric cancer cells [59], the loss of EMILIN-2 expression may also display a direct effect of gastric cancer cell growth and progression. Finally, the analyses of EMILIN-1 revealed that in some cases tumor samples were characterized by an altered organization of the molecule in the lamina propria. It is possible that this altered deposition of EMILIN-1 may lead to an impairment of the formation of the lymphatic vasculature as described in other tissues [51,52,53]. The co-localization analysis revealed that the samples displaying the lower expression of EMILIN-2 were also characterized by a major disorganization of the EMILIN-1 fibers, suggesting that the loss of EMILIN-2 expression may also affect the organization of EMILIN-1. In fact EMILIN-1 and EMILIN-2 have been shown to interact in a complex way during microfibril assembly [60]. As an important regulator in the tumor microenvironment, EMILIN-2 gene is methylated in a number of tumors [61], thus it is possible that patients displaying low EMILIN-2 expression may be characterized by high levels of EMILIN-2 gene methylation. It is also possible that the loss of EMILIN-2 may be due to increased degradation of the molecule, since the ECM remodeling is known to occur during cancer progression. Given that both blood and lymphatic vessels represent a route for metastatic cell dissemination, the altered expression of EMILIN-2 and EMILIN-1 may significantly impact of the progression of gastric tumors. Taken together our results demonstrate that the molecules of the EDEN family are highly expressed in gastric tissue and that the loss of their expression observed in tissue samples may significantly affect the efficacy of the treatments as well as tumor progression. Thus, we strongly consider that to carry out these analyses in a higher number of patients could lead to the development of new tools to predict therapy efficacy.

## 4. Materials and Methods

### 4.1. Cell Cultures 

Human Umbilical Vein Endothelial Cells (HUVEC) were isolated from the human umbilical cord vein, as previously described [41] and cultured in endothelial cell medium (ScienCell Research Laboratories, Inc., San Diego, CA, USA). Human gastric adenocarcinoma AGS cells (ATCC^®^ CRL-1739™) were cultured in F12K medium (Kaighn’s Modification of Ham’s F-12 Medium, ATCC^®^, Manassas, VA, USA) containing 10% FBS (GIBCO, Milan, Italy) and antibiotics. 

### 4.2. Tube Formation Assays

50 μL of MATRIGEL^®^ GFR (Franklin Lakes, NJ, USA) were added to each well of a 96 well-plate and allowed to solidify for 30 min at 37 °C. HUVEC cells were starved for 4 h, treated with trypsin, harvested and resuspended in AGS-conditioned media containing or not 5 μg/mL of recombinant EMILIN-2 (conditioned media were obtained culturing AGS cells in 0.5% FBS-ECM medium for 48 h; recombinant EMILIN2 was produced as previously shown [40]). HUVEC cells were then seeded onto the layer of MATRIGEL^®^ (3.0 × 10^4^ cells/well) and cultures placed in the cell incubator (37 °C and 5% CO_2_) of a Leica Time Lapse AF6000LX workstation (Leica Microsystems GmbH, Wetzlar, Germany) interfaced with the Leica Application Suite (LAS) software. Images were automatically taken every 15 min for an 8 h period of observation and analyzed using online WimTube software (Wimasis Image Analysis, Ibidi, Onimagin Technologies SCA, Cordoba, Spain) to measure the number of total tubes, total branching points and total loops or the length of formed tubes.

Another set of tubulogenesis experiments with AGS-derived media was performed, with the same procedures, using HUVEC cells transduced with a Multimerin-2 specific siRNA adenoviral construct (siMMRN2) [41,42]; cells transduced with the scrambled vector (siCNTR) were included as control.

### 4.3. Patients

For this study we enrolled 51 patients based on the presence of locally advanced gastric cancer and agreement to sign the informed consent form to undergo pCLE endomicroscopy analyses. In the present manuscript, as an example, we reported immunofluorescence analyses from one patient displaying high levels of EMILIN-2 loss in cancer tissue and one displaying only modest loss of EMILIN-2 loss. The study was conducted in accordance with the Declaration of Helsinki, and the protocol was approved by the Institutional Board of CRO-IRCCS, National Cancer Institute of Aviano (PN), Italy (IRB no. CRO-2014-03, approval date: 22 September 2014). Laboratory and pathological results were collected by means of the hospital database.

### 4.4. Endoscopy Procedures and pCLE Analyses

The patients were examined before any radio/chemotherapeutic or surgical intervention. During the gastric endoscopy procedure (Olympus series180, Segrate, Italy), pCLE analyses were carried out by means of the GastroFlex UHD probe (Cellvizio, Mauna Kea Technology, Paris, France) as previously described [56]. Normal and neoplastic mucosa were examined and images and sequences were recorded within the first 10 min following i.v. injection of fluorescein (5 mL of a 10% solution). To ensure high video quality, pCLE images were collected at 12 frames per second, allowing a direct visualization on a single erythrocyte scale. pCLE recordings were carried out for 3 min resulting in a real-time imaging of more than 2000 frames and were functional to reconstruct the scanned panoramas of the mucosa. The images were reviewed by a single investigator that was blinded to any clinical, endoscopical, or histopathological information. Intra-tumoral angiogenesis was evaluated in terms of extent and quality of the vessels; the angiogenic score was determined based on the presence of tortuous and large sized vessels, the vessels’ leakage and the presence of defective flow. At the end of the endoscopic examination, the conventional bioptic samples were obtained by macrobiopsy (COOK Medical, Bloomington, IN, USA) and the specimens were submitted for pathological examination. From each patient additional samples were collected, included in the optimal cutting temperature compound (OCT), snap-frozen and stored for further immunofluorescence analysis. 

### 4.5. Immunofluorescence

For immunofluorescence analyses on tumor samples, serial cryostatic sections (7 μm) were collected on positively charged slides (BDH Superfrost^®^ Plus; VWR International S.r.l., Milan, Italy), air dried at room temperature (RT) and fixed with PFA for 15 min. After washing with phosphate-buffered saline (PBS), the slices were incubated with 0.5% Triton X-100 in PBS for 5 min at RT, saturated with 1% BSA-10% normal goat serum (DAKO) in PBS for 1 h at RT, and stained ON at 4 °C with the appropriate antibodies. Next, the samples were washed with PBS and incubated with the appropriate secondary antibodies and TO-PRO3 to stain the nuclei for 1 h at RT. After washing with PBS, the slides were mounted in Mowiol containing 2.5% (*w*/*v*) of 1,4-diazabicyclo-(2,2,2)-octane (DABCO). Images were acquired with a Leica TCS SP8 Confocal system (Leica Microsystems Heidelberg, Mannheim, Germany), using the Leica Confocal Software (Leica Application Suite X version 3.1.5.16308). Fluorescence intensity and quantification was evaluated by means of the Volocity software Version 6.1.1 (PerkinElmer Inc., Waltham, MA, USA).

The polyclonal anti-Multimerin-2 and anti-EMILIN-2 antibodies were produced in our laboratories as previously described [38,43]. The mouse anti-human gC1q (clone 1H2) antibody was obtained as already reported [51,62]. The monoclonal anti-human CD31 antibody was from Invitrogen (Milan, Italy). The secondary antibodies conjugated with Alexa Fluor 488, 546 and TO-PRO-3 were from Invitrogen (Milan, Italy).

### 4.6. Statistical Analyses

Statistical analyses were performed with the SigmaPlot version 11 software and the values represent the mean ± standard deviation. The statistical significance of the differences was determined by the two-sided Student’s *t*-test for the comparisons between two groups. For all the evaluations reported in the manuscript, the investigators were blinded. All the measurements were included for the statistical analyses and differences were considered statistically significant when *p* ≤ 0.05.

## 5. Conclusions

In conclusion, in this study we characterized the expression of EMILIN-1, Multimerin-2 and EMILIN-2 in the gastric normal mucosa and in gastric cancer. We provided evidence that the expression of Multimerin-2 and EMILIN-2 are significantly altered in a number of gastric cancer patients. Given the impact of these two molecules in angiogenesis the loss of their expression may significantly impact on tumor progression and therapy efficacy. Thus, the evaluation of their expression may be useful to predict the patients’ outcome.

## Supplementary Materials

Supplementary materials can be found at http://www.mdpi.com/1422-0067/19/12/3983/s1.
