# Supplementary material for: Loss of Multimerin-2 and EMILIN-2 Expression in Gastric Cancer Associate with Altered Angiogenesis
Source: Int J Mol Sci. 2018 Dec 11;19(12):3983. doi: 10.3390/ijms19123983 (PMC6321373; doi:10.3390/ijms19123983)
Supplement: Supplementary file 1 [file ijms-19-03983-s001.zip › ijms-393094-SI/ijms-393094 Supplemental material for proof/Figure S1.pdf]

Supplementary Figure S1

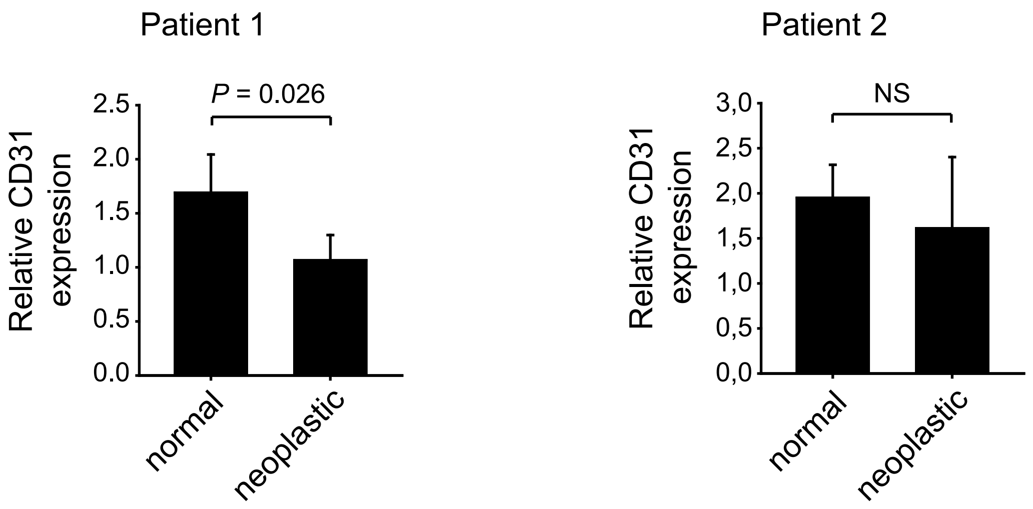

Figure S1: Graphs reporting the analysis of CD31 staining in normal and gastric cancer biopsies from patient 1 and 2, as indicated. Values represent the mean ± SD; P values were obtained using the paired Student's t-test.
