# Supplementary material for: Loss of Multimerin-2 and EMILIN-2 Expression in Gastric Cancer Associate with Altered Angiogenesis
Source: Int J Mol Sci. 2018 Dec 11;19(12):3983. doi: 10.3390/ijms19123983 (PMC6321373; doi:10.3390/ijms19123983)
Supplement: Supplementary file 1 [file ijms-19-03983-s001.zip › ijms-393094-SI/ijms-393094 Supplemental material for proof/Supplemetal Video S1 .pdf]

Supplemetal Video S1 showing the vasculature of a gastric cancer patient characterized by an angiogenic score 3 according to the Cannizzaro/Spessotto scale as detected by pCLE. Defective flow is clearly visible.
